# Gene Editing Correction of a Urea Cycle Defect in Organoid Stem Cell Derived Hepatocyte-like Cells

**DOI:** 10.3390/ijms22031217

**Published:** 2021-01-26

**Authors:** Mihaela Zabulica, Tomas Jakobsson, Francesco Ravaioli, Massoud Vosough, Roberto Gramignoli, Ewa Ellis, Olav Rooyackers, Stephen C. Strom

**Affiliations:** 1 Department of Laboratory Medicine, Karolinska Institute, 141 52 Stockholm, Sweden; mihaela.zabulica@ki.se (M.Z.); tomas.jakobsson@ki.se (T.J.); roberto.gramignoli@ki.se (R.G.); 2 Department of Experimental, Diagnostic and Specialty Medicine, University of Bologna, 40 138 Bologna, Italy; francesco.ravaioli2@unibo.it; 3 Department of Regenerative Medicine, Cell Science Research Centre, Royan Institute for Stem Cell Biology, Tehran 16635-148, Iran; masvos@royaninstitute.org; 4 Department of Clinical Sciences Intervention and Technology, Karolinska Institute, 141 86 Stockholm, Sweden; ewa.ellis@ki.se (E.E.); olav.rooyackers@ki.se (O.R.)

**Keywords:** iPSC, hepatocytes, disease modelling, genome editing, CRISPR, urea cycle

## Abstract

Urea cycle disorders are enzymopathies resulting from inherited deficiencies in any genes of the cycle. In severe cases, currently available therapies are marginally effective, with liver transplantation being the only definitive treatment. Donor liver availability can limit even this therapy. Identification of novel therapeutics for genetic-based liver diseases requires models that provide measurable hepatic functions and phenotypes. Advances in stem cell and genome editing technologies could provide models for the investigation of cell-based genetic diseases, as well as the platforms for drug discovery. This report demonstrates a practical, and widely applicable, approach that includes the successful reprogramming of somatic cells from a patient with a urea cycle defect, their genetic correction and differentiation into hepatic organoids, and the subsequent demonstration of genetic and phenotypic change in the edited cells consistent with the correction of the defect. While individually rare, there is a large number of other genetic-based liver diseases. The approach described here could be applied to a broad range and a large number of patients with these hepatic diseases where it could serve as an in vitro model, as well as identify successful strategies for corrective cell-based therapy.

## 1. Introduction

Liver is an organ performing a diverse repertoire of metabolic processes, including ammonia metabolism and urea production through the urea cycle. Mutations in any of the genes encoding the six enzymes or two mitochondrial transporters that constitute the urea cycle could lead to urea cycle disorders (UCD). In the case of UCD, excess nitrogen (in the form of ammonia) accumulates in the body and may result in irreversible neurological and intellectual impairment, growth retardation and hyperammonemic episodes, which can prove fatal if not rapidly corrected [1].

The most common UCD is ornithine transcarbamylase (OTC) deficiency (OTCD), which affects 1 in 56,000 individuals [2]. The disease is inherited in an X-linked manner; therefore, affected males frequently have more severe symptoms than females. Criteria for diagnosis of OTCD affected patients have been established and include elevated ammonia levels and detection of mutations in the OTC gene, decreased enzyme activity in the liver or family history of the disorder along with elevated urinary orotate.

Cornerstones of OTCD treatment are restriction of protein intake supplemented with pharmaceutical intervention activating alternative pathways for the excretion for ammonia clearance. However, these measures are not always sufficient to maintain a patient’s metabolic balance. The only definitive treatment of OTCD is orthotopic liver transplantation, which involves the surgical removal of the entire organ and replacement with a healthy donor liver. Cell-based therapy is also considered a treatment for patients with metabolic diseases [3,4,5,6]. However, it is less frequent due to limited availability of donor hepatocytes, low proliferative capacity and inability of primary hepatocytes to maintain mature functions in vitro [7,8,9]. Recent studies have also reported that the survival of donor hepatocytes after transplantation can be affected by the immune-mediated rejection of the injected cells [6,10].

Additionally, among the restrictions for testing and identification of novel therapeutics for the treatment of liver diseases is the inability to model the hepatic function and phenotype in vitro. Immortalized cancer cell lines are widely used. However, they do not express many hepatic functions at adequate levels that would resemble primary hepatocytes, have dysfunctional apoptotic pathways due to their carcinogenic nature and present restricted genotypic variability because of their origin [11]. Another potential source of cells for in vitro drug testing are primary hepatocytes, which are currently considered the gold standard and have been shown to biochemically and morphologically resemble healthy liver cells in vivo to a satisfactory degree [12,13]. Yet, they are characterized by disadvantages including de-differentiation and gradual loss of hepatic functions after plating, as well as inability to proliferate and expand [14,15].

The discovery of induced pluripotent stem cells (iPSC) by the forced expression of transcription factors (Yamanaka factors, Oct4, Sox2, Klf4 and cMyc) [16], and the demonstration that they differentiate into all three germ layers, raised enthusiasm for generating an almost inexhaustible cell source for cell replacement therapies and in vitro drug screening. A prominent advantage of iPSC, over embryonic stem cells (ESC), is the ability to be derived from adult human cells with various genetic backgrounds including patients with rare genetic disorders. Additionally, iPSC avoid the ethical controversies of ESC, and their use in an autologous transplant could potentially evade life-long immunosuppressive treatment.

During the past two decades, a plethora of studies have aimed to generate mature and functional human liver cells. Currently used hepatic differentiation protocols attempt to mirror in vitro the natural events that occur during normal development. The vast majority of protocols are based on the utilization of growth factors and small molecules imitating natural processes, starting from ESC in the blastula, to definitive endoderm, early hepatic progenitors, hepatoblasts, fetal-like and finally more mature hepatocytes.

Additional technological developments in the gene engineering field based on clustered regularly interspaced short palindromic repeat (CRISPR)/CRISPR-associated (Cas) systems [17,18,19] have facilitated the quick, efficient and inexpensive manipulation of the genome. With these technologies, limitations of conventional strategies of inserting a transgene with a viral vector can be overcome. CRISPR/Cas technology has been applied to a broad range of cell types, including human iPSC, that might be a useful source of an autologous graft upon differentiation into the functional mature cell type of interest. However, in genetic disorders, the generated iPSC would still carry the mutation causing the disease. Therefore, a combination of cell reprogramming and gene engineering technologies could raise the potential for the generation of a cell source for autologous therapies.

In this study, we generated iPSC from a patient with severe OTCD carrying a mutation which abolishes almost completely the enzyme activity, successfully corrected the genetic mutation through homology-directed repair (HDR) and characterized the phenotype of edited iPSC-derived organoid hepatocyte-like cells (HLC). Here, we demonstrate the first OTCD iPSC models and their corrected counterparts, as well as their differentiation into organoid HLC.

## 2. Results

### 2.1. Disease-Causing Variant Identification and Transcript Characterization

**Figure 1:**
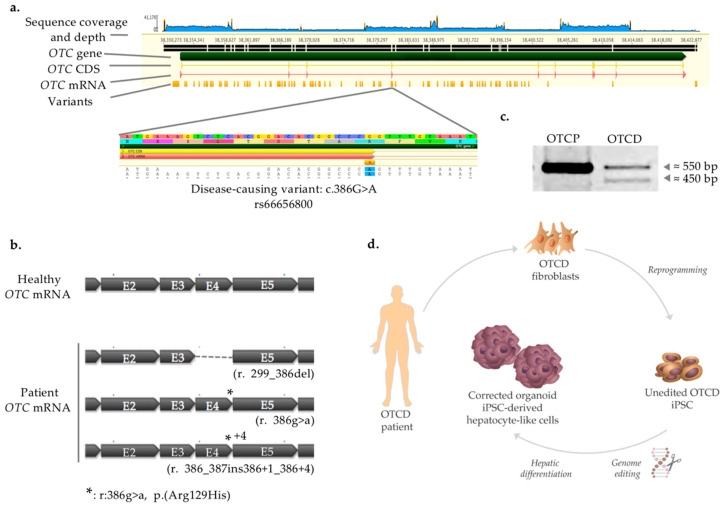
Mutation identification and study overview. (**a**) *OTC* gene sequence alignment in OTC-deficient (OTCD) patient to reference gene. Sequencing coverage and depth, *OTC* gene, coding sequence (CDS), mRNA and variants identified after alignment of *OTC* gene in OTCD patient to reference gene (NCBI ID: 5009) are shown. The genomic region containing the single nucleotide polymorphism (SNP, rs66656800) causing the disease is presented in the bottom panel (c.386G>A). (**b**) Representation of *OTC* transcript in healthy (OTC-proficient, OTCP) hepatocytes and OTCD patient. Three different *OTC* transcripts are present in patient’s hepatocytes: skipping of exon 4 (r.299_386del), elongation of exon 4 with the first 4 bp of intron 4 (r.386_387ins386+1_386+4) and the full length of transcript with exon 4 harboring the mutation (r. 386g>a). Grey boxes represent introns (E2, E3, E4, E5). *: Mutation r.386g>a on RNA level which results in Arg129His substitution on protein level. (**c**) Amplification of *OTC* transcript. Amplification of *OTC* transcript spanning exons 1 to 5 was performed in normal (OTCP) and OTCD hepatocytes. OTCD appeared to have bands of two different lengths, around 550 (wild-type) and 450 bp. (**d**) Schematic diagram depicting the overview of the study. Fibroblasts from the OTCD donor were reprogrammed into induced pluripotent stem cells (iPSC). Thereafter, the cells were submitted to genome engineering to correct the disease-causing variant. Finally, cells were differentiated into hepatocyte-like cells through organoid formation and were phenotypically characterized (Illustration was partly generated with images from © Adobe Stock, Mountain View, CA, USA).

Liver fibroblasts from a 9 month old male with severe OTCD who underwent orthotopic liver transplantation were isolated and cultured in feeder-free conditions. In order to identify the disease-causing variant, long-range PCR was used to amplify the whole *OTC* gene, covering both introns and exons. Amplicons were sequenced and aligned to the reference gene on NCBI (ID: 5009) (Figure 1a). Out of 120 variants identified, one has been previously reported as pathogenic (c. 386G>A, rs66656800) and extensively characterized [20]. Specifically, three different *OTC* transcripts were described as present in the patient’s hepatocytes: skipping of exon 4 (r.299_386del), elongation of exon 4 with the first 4 bp of intron 4 and spliced by a cryptic splice site in intron 4 (r.386_387ins386+1_386+4), and finally the full length of transcript naturally spliced containing exon 4 and harboring the mutation (r. 386g>a) (Figure 1b). In order to validate that the same pattern is observed in OTCD cells, we amplified the *OTC* transcript in primary hepatocytes derived from the OTCD patient, as well as from “normal”, OTC-proficient (OTCP) hepatocytes, serving as positive control. Indeed, the analysis revealed the presence of transcripts of two lengths in the OTCD patient, around 550 (wild-type length) and 450 bp (Figure 1c). The length difference of approximately 100 bp could be predicted since exon 4, approximately 100 bp long, is omitted in two out of three messenger RNAs. Additionally, the difference of 4 bp between two transcripts makes it impossible to separate them on the agarose gel; therefore, only two bands can be evident (Figure 1c).

The study overview is presented in Figure 1d. Briefly, somatic cells derived from the liver of an OTCD patient were reprogrammed into iPSC and genetically engineered to correct the mutation causing the disease. Thereafter, iPSC were differentiated into HLC organoid, and the phenotype was characterized in vitro.

### 2.2. Generation and Characterization of Patient-Derived iPSC

**Figure 2:**
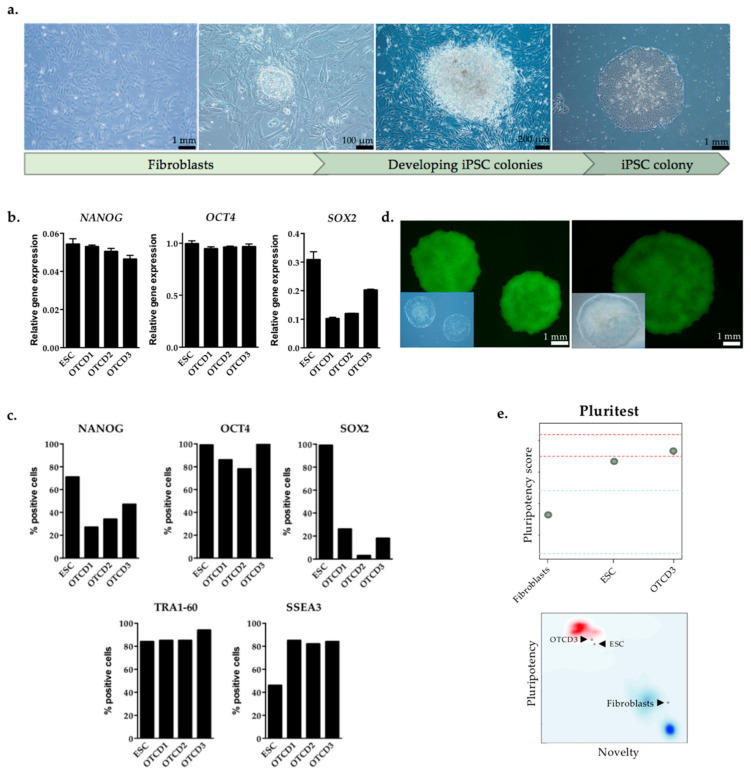
Generation and characterization of patient OTCD iPSC. (**a**) Generation of iPSC. Progressive stages of patient fibroblast reprogramming into iPSC are presented, starting from fibroblasts (**left**), moving into emerging iPSC colonies (**middle**), and eventually obtaining typical iPSC colony with characteristic features including sharp edges, round shape and homogeneous cell population (**right**). Scale bars are indicated in the respective images. (**b**) Expression profile of OTCD iPSC clones. Three different iPSC clones obtained from reprogramming OTCD fibroblasts, denoted as OTCD1, OTCD2 and OTCD3, were characterized for gene expression profile of pluripotency markers (*NANOG*, *OCT4* and *SOX2*). The expression was normalized to endogenous control gene (*PPIA*) and compared to the respective levels in embryonic stem cells (ESC). Technical replicates n = 3. (**c**) Protein levels of iPSC clones. Three different iPSC clones obtained from reprogramming of OTCD fibroblasts, denoted as OTCD1, OTCD2 and OTCD3, were characterized for protein expression profile of pluripotency markers (NANOG, OCT4, SOX2, TRA-1-60, SSEA3) through flow cytometry and compared to the respective levels in ESC. (**d**) Alkaline phosphatase activity staining as a pluripotent marker. Representative pictures of alkaline phosphatase activity staining on generated iPSC colonies from OTCD patient cells. Scale bars are indicated in the respective images. (**e**) PluriTest. Pluripotency scores (**upper panel**) of fibroblasts (negative control), ESC (positive control) and OTCD3 iPSC clone were assessed through PluriTest. Red and blue areas in the **lower panel** refer to pluripotent and differentiated profiles, respectively, within SCM2 matrix data set. Fibroblasts, ESC and OTCD3 iPSC clones are indicated with arrows. n = 1.

Liver fibroblasts, derived from the OTCD patient, were cultured in feeder-free conditions and transduced with Sendai virus, a non-integrating vector, expressing the Yamanaka transcription factors. Approximately three weeks post-transduction, emerging iPSC colonies with typical morphology (flat, densely packed colonies with sharp, round edges) could be observed, as shown in Figure 2a (right). Six iPSC clones were isolated, and of those, three were chosen for the study based on growth characteristics and markers of pluripotency (clones are denoted as OTCD1, OTCD2 and OTCD3). Pluripotency markers in OTCD clones were measured through gene expression (Figure 2b) and protein levels (Figure 2c) and compared to the respective levels in an ESC clone. IPSC clones expressed *NANOG* and *OCT4* to a similar extent as ESC, while lower levels of SOX2 (Figure 2b). Moreover, iPSC clones displayed a high level of SSEA3 and equal amount of OCT4 and TRA-1-60 proteins, compared to ESC, but lower NANOG and SOX2 (Figure 2c). Additionally, iPSC colonies stained positive for alkaline phosphatase, a characteristic enzyme of rapidly replicating cells and marker of pluripotency (Figure 2d).

Another method for examining gene expression but on a more global scale is PluriTest, which compares the transcriptomic profiles of generated iPSC clones to well-documented ESC or iPSC. PluriTest is an online tool which assesses pluripotency and novelty scores based on a holistic transcriptomic profile. Novelty score indicates patterns of gene expression consistent with a differentiated state, while pluripotency score shows the expression profile associated with pluripotency and thereby distant from the profile of differentiated cells [21,22]. As described later in this article, OTCD3 clone was selected for genome engineering and hepatic differentiation due to its higher propensity to differentiate into endoderm lineage compared to OTCD1 and OTCD2 iPSC. Thus, only OTCD3 clone was subjected to PluriTest analysis. OTCD3 had high pluripotency and low novelty scores when compared to mature differentiated cells, in this instance fibroblasts (negative control). Finally, the selected iPSC clone was situated in close proximity to ESC (positive control) in the MMC2 matrix, as they showed similar novelty and pluripotency scores (Figure 2e).

### 2.3. CRISPR/Cas-Mediated Genome Editing of Patient iPSC

**Figure 3:**
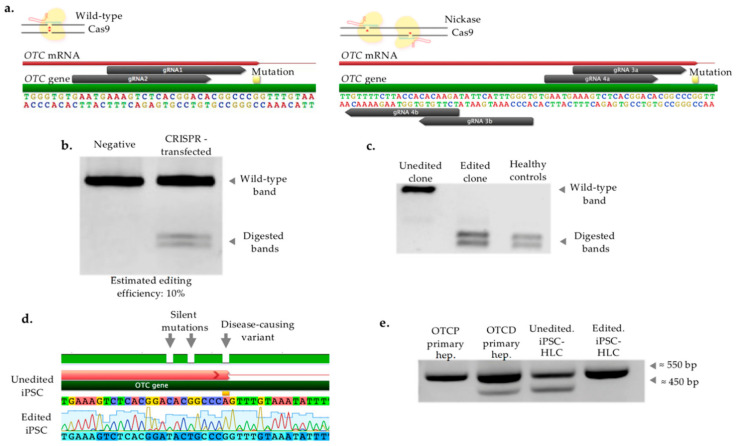
CRISPR/Cas9-mediated gene targeting of patient’s OTCD iPSC. (**a**) Genome engineering strategies. Single guide RNA (gRNA1, gRNA2) (**left panel**) and dual gRNA (combination of gRNA3a with gRNA3b, and gRNA4a with gRNA4b) (**right panel**) approaches were used to achieve the best editing efficiency. Single guides were delivered along with wild-type Cas9, while dual guides with nickase Cas9. Position of mutation respective to gRNAs is marked in the figure. (**b**) Assessment of cleavage efficiency. Editing efficiency was estimated based on intensities of wild-type and cleaved bands following restriction enzyme digestion. Negative: untransfected cells. CRISPR-transfected: cells transfected with Cas9 and gRNA. (**c**) Restriction enzyme assay—clone screening. A total number of 54 iPSC clones were serially isolated, expanded and screened. Screening was conducted with restriction enzyme digestion assay which would digest only successfully corrected DNA sequence of targeted region. One iPSC clone was edited on both alleles having only digested bands, similar to the healthy control (positive control). A representative unedited clone is also shown. (**d**) Sequencing of genomic DNA of unedited and edited clones. The genomic region of interest containing the disease-causing variant was Sanger-sequenced in unedited and edited iPSC clones. Three base pairs were identified as differences between the parental and the engineered cells (indicated with arrows), as expected. Two of those are silent mutations which were deliberately introduced to increase the correction efficiency, and one is the mutation intended to be edited to correct OTC deficiency. (**e**) Investigation of *OTC* transcript. Amplification of OTC transcript spanning from exon 1 to exon 5 was performed in OTC-proficient (OTCP) primary and OTCD primary hepatocytes, as well as in unedited and edited iPSC hepatocyte-like cells (iPSC-HLC). One wild-type band was presented in cells correctly expressing the gene (OTCP primary hepatocytes and edited iPSC-HLC), while two bands (wild-type and shorter band due to exon skipping) in genetically defected cells (OTCD primary hepatocytes and unedited iPSC-HLC).

Guide RNA (gRNA) was designed and constructed in order to induce double-stranded DNA breaks proximal to the locus to be genetically altered. In total, two single gRNAs were selected to be used with wild-type Cas9, as well as two pairs of gRNA to be transfected with nickase Cas9 (D10A), as illustrated in Figure 3a. Sequences of guides and DNA donor templates utilized are provided in Tables S1 and S2, respectively. Editing efficiency was evaluated with a restriction enzyme assay which digests DNA sequences that have correctly undergone homology-directed repair (HDR). The combination of wild-type Cas9 and gRNA2 was measured to give approximately 10% editing efficiency and was used for the study (Figure 3b). This was further validated with Interference of CRISPR Edits (ICE, Synthego, Redwood City, CA, USA) web tool—around 8.4% (Figure S1).

Subsequently, single cell clonal expansion was performed. In total, 54 clones were isolated, expanded and subjected to restriction digestion enzyme assay. One edited clone was identified to be fully digested, the same as normal OTCP cells (Figure 3c). DNA sequencing of parental, unedited iPSC and edited iPSC confirmed the genetic correction. Specifically, when sequences from unedited and edited cells were aligned, three single bp differences were identified, as expected, with one being the mutation intended to be repaired (G > A) and two silent mutations (GAC > GAT, ACG > ACT), deliberately introduced into the donor template to increase CRISPR efficiency by avoiding the cleavage of donor template and re-cleavage of already-edited DNA site (Figure 3d). Moreover, we investigated the *OTC* transcripts in healthy OTCP and OTCD primary hepatocytes, as well as unedited and edited iPSC-HLC by amplification of exons 1 to 5 (hepatic differentiation is extensively described and results are shown in the next sections, while *OTC* transcripts from differentiation experiments are presented here for the purpose of verifying the correction). Figure 3e confirms the presence of two bands (three *OTC* transcripts) in cells incorrectly expressing the gene (patient OTCD primary hepatocytes and unedited iPSC-HLC), while only the wild-type band in OTCP primary hepatocytes and edited iPSC-HLC (Figure 3e). The lengths of transcripts were as expected—550 bp for the wild type, and the shorter band was around 450 bp because of exon 4 skipping. Furthermore, sequencing of *OTC* transcript confirmed the restoration of exon 4 in edited iPSC-HLC, and consequently, successful gene correction (Figure S2).

The expression of pluripotency markers was re-assessed in unedited and edited iPSC. *NANOG* and *OCT4* were found to be at similar levels, while *SOX2* higher in edited cells (Figure S3).

### 2.4. Differentiation of iPSC into Organoid iPSC-HLC

It is widely accepted that stem cell clones exhibit differences in the propensity to differentiation into specific cell types, depending on various factors including cell source, methylation landscape or differences between clones [23,24,25,26]. Therefore, we induced iPSC clones OTCD1, OTCD2 and OTCD3 into definitive endoderm with two different protocols (DE protocol A and DE protocol B) and evaluated the gene expression of pluripotency (*NANOG*, *OCT4* and *SOX2*), and definitive endoderm markers (*SOX17*, *FOXA2* and *CXCR4*) of generated cells. It was evident that endoderm cells generated from OTCD3 had pluripotency genes shut down with both protocols (Figure S4a), while definitive endoderm markers substantially increased (Figure S4b). Conclusively, we decided to use the OTCD3 clone for genome engineering and differentiation experiments, since it showed the highest differentiation capacity into the endoderm lineage that hepatocytes belong to.

**Figure 4:**
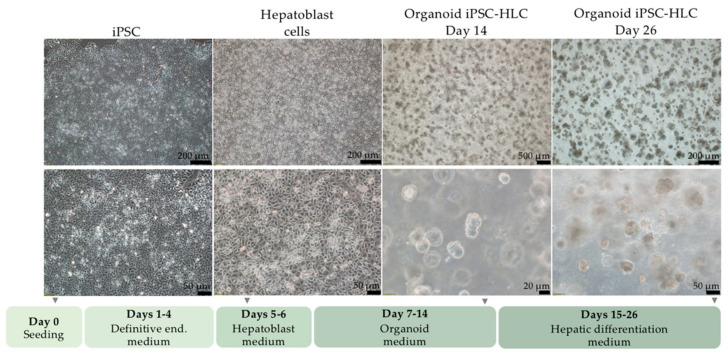
Stages of hepatic differentiation. Representative pictures of iPSC differentiation towards hepatoblast, and eventually organoid iPSC hepatocyte-like cells (iPSC-HLC) are shown at different magnifications. Synopsis of differentiation protocol is shown at the bottom of the figure. Scale bar indicated in each image.

Next, we sought to examine whether *OTC* gene editing resulted in functional correction in the gene-repaired cells. Since the urea cycle is typically expressed in hepatocytes, we differentiated gene-edited and parental unedited patient iPSC into HLC organoids. Optimization steps were performed to test the optimal seeding cell number and coating protein resulting in Matrigel and 10^5^ cells as optimal conditions (Figure S5). The hepatic differentiation protocol consisted of four sequential stages lasting 26 days in total. An overview of the hepatic differentiation protocol and representative pictures from each stage are shown in Figure 4. On day 7 of differentiation, definitive endoderm cells were seeded in Matrigel to generate organoid structures. Few organoid bodies eventually seeded to the bottom of the culture plate, facilitating the visualization of the morphology of obtained iPSC-HLC by phase microscopy. Polygonal morphology of the cells was observed as lower nucleus to cytoplasm ratio, while differentiation advanced. Spare binucleated iPSC-HLC with enriched lipid vesicles were observed, characteristics of primary hepatocytes (Figure S6).

### 2.5. Gene Expression of Organoid iPSC-HLC

**Figure 5:**
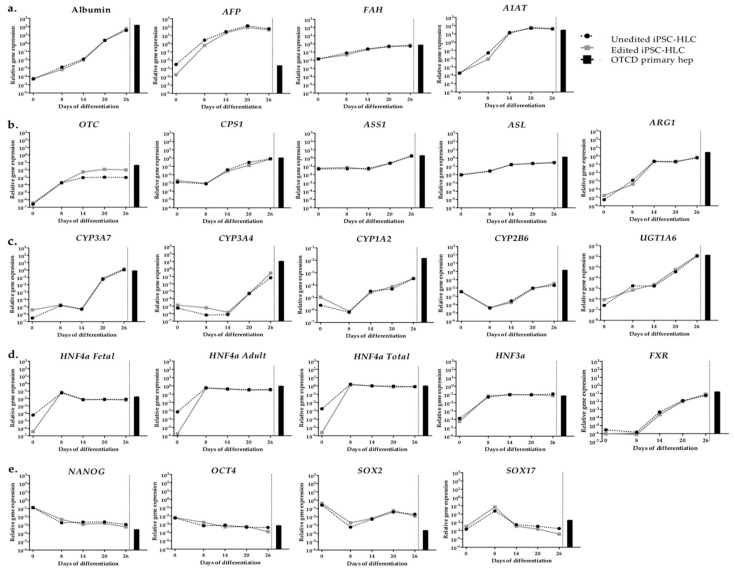
Gene expression profiling of organoid iPSC hepatocyte-like cells (HLC) organoids. Expression of genes for liver-specific plasma proteins and metabolic enzymes (**a**), urea cycle proteins (**b**), phase I and II conjugation proteins (**c**), transcription factors (**d**) and pluripotency genes (**e**) was measured at different time points of differentiation protocol. Dashed and continuous lines show unedited and edited iPSC-HLC organoids, respectively. Black bar indicates the level of expression of the respective gene in primary OTCD hepatocytes from the same patient. Expression levels were normalized to endogenous gene (*PPIA*). Technical replicates n = 2.

In order to evaluate the maturation and essential hepatic functions of unedited and edited iPSC-HLC, RT-qPCR was performed on 26 genes, including liver-specific proteins; urea cycle genes; phase I and phase II genes; transcription factors; and pluripotency, definitive endoderm genes and transporters (Figure 5 and Figure S7). Gene expression was evaluated at different stages (day 0: iPSC, day 8: definitive endoderm, day 14: initiation of organoid formation, day 20: early iPSC-HLC, and day 26: late iPSC-HLC) and compared to the respective gene expression in OTCD primary hepatocytes from the same patient. Generally, the expression of each gene changed throughout the differentiation suggesting occurrence of transition from iPSC to endoderm, towards hepatoblast cells and finally into iPSC-HLC. Expression of hepatic markers such as albumin (*ALB*) and alpha-fetoprotein (*AFP*) are illustrated in Figure 5a. Albumin increased approximately six logs from pluripotency to the hepatic state, while *AFP* reached a plateau at day 14 and maintained this level until the end of experiment suggesting preservation of fetal-like characteristics. Other liver-related genes, such as fumarylacetoacetate hydrolase (*FAH*), alpha-1 antitrypsin (*A1AT*) and urea cycle genes were measured at the end of differentiation at levels similar to OTCD primary hepatocytes (with the exception of *OTC*) (Figure 5a,b). As shown in Figure 5c,d, phase I and II genes and transcription factor expression increased over time. Finally, the differentiation of iPSC into different cell types requires the repression of pluripotency genes. Therefore, *NANOG*, *OCT4*, *SOX2* and *SOX17* were analyzed with *NANOG*, *OTC4* and *SOX17* decreasing over time and reached primary hepatocyte levels, while *SOX2* decreased but remained around two logs higher than the levels in primary hepatocytes (Figure 5e). Transporters *BCRP*, *BSEP* and *NTCP* were evaluated and found to exceed the respective levels observed in primary OTCD hepatocytes (Figure S6).

Remarkably, there were no obvious differences between genetically edited and unedited iPSC-HLC in all examined genes at all time points, except from *HNF4a* at day 0 and *OTC* after day 8. We reasoned that the reason for having constant, stable differences in OTC expression between repaired and non-repaired iPSC-HLC is the instability of the mutant transcript in unedited iPSC-HLC.

### 2.6. Phenotypic Characterization In Vitro

**Figure 6:**
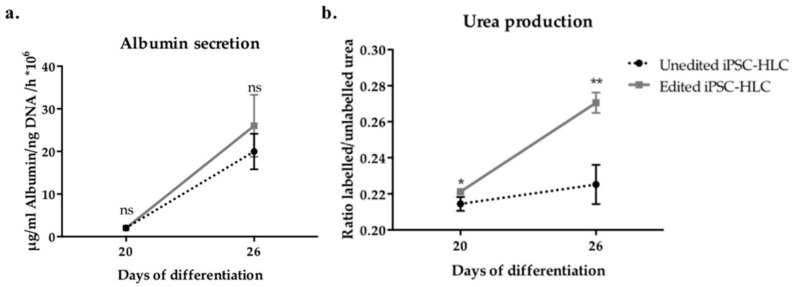
Albumin secretion and ^15^N incorporation into urea. (**a**) Albumin secreted by unedited and edited iPSC hepatocyte-like cell (iPSC-HLC) was measured at days 20 and 26 of hepatic differentiation. Averages and errors are shown as means and standard deviation. Mann–Whitney *U* test was used for statistical analysis. Biological replicates n = 3. Ns: Not significant. (**b**) Incorporation of labelled ^15^N into urea, which indicates the urea produced through the urea cycle, by unedited and edited iPSC-HLC, was quantified with mass spectrometry at days 20 and 26 of hepatic differentiation. Biological replicates n = 6. Mann–Whitney *U* test was used for statistical analyses. Ns: Not significant, *p* > 0.05, *: *p* ≤ 0.05, **: *p* ≤ 0.01.

Albumin is an abundant protein in human plasma produced by the liver. We sought to quantify the secreted amounts of albumin by unedited and edited iPSC-HLC on days 20 and 26. As shown in Figure 6a, albumin secretion progressively increased with no statistical differences between unedited or edited cells (*p* > 0.05).

Additionally, urea production and secretion by differentiated cells was investigated. Since iPSC-HLC are derived from hepatic fibroblasts isolated from an OTCD patient, we anticipated deregulated urea cycle activity in unedited iPSC-HLC. Additionally, urea cycle activity requires the coordinated expression and activity of multiple proteins, and the iPSC-HLC generated in this study did not express all of them at levels comparable with primary hepatocytes (Figure 5b). Therefore, if editing was successful, one could expect an improvement in urea production in edited iPSC-HLC, but not a full restoration of normal hepatic activity. Figure 6b shows a gradual increase in urea production and the differences between unedited and edited iPSC-HLC.

### 2.7. Off-Target Mutagenesis Analysis

Unspecific and unintended genetic alterations are common concerns when applying genome editing technologies. Therefore, we performed whole genome sequencing of genomic DNA extracted from unedited and edited iPSC. Genotypic differences between the two clones were extracted and searched for the target gRNA sequence as a query against the extracted references. The variant list obtained is shown in Table S3. Specifically, three potential off-target sites were identified, in chromosomes 5, 11 and 13. Since none of these potential off-target sites had a protospacer adjacent motif (PAM) sequence, and all had 4–6 bp mismatches with the gRNA sequence used in the study, we concluded that none of them was an off-target from CRISPR application. Additionally, even in the case that these sites were unintentionally altered, they are in intronic or intergenic regions, and most likely would not have significant biologic consequences. Finally, among differences identified between unedited and edited clones were 3-point mutations in the *OTC* gene (patient mutation and two silent mutations deliberately introduced) which confirm successful editing (Table S4).

## 3. Discussion

The aim of this study was to present a useful model system to identify, genetically correct and verify a change in the phenotype of cells isolated from a patient with a rare liver disease. Due to low OTCD incidence, primary hepatocytes are frequently not available; thus, a surrogate model was generated with iPSC. The hypothesis tested was that the iPSC which contain the rare mutation and hepatic defect could be employed to identify successful editing procedures, and when the edited or unedited cells were successfully differentiated into organoid HLC, a difference in the phenotype could be identified. In the study reported here, iPSC were generated from a patient with severe OTCD. They were subsequently genetically edited to correct the mutation and differentiated into iPSC-derived HLC. When compared to the unedited cells, the edited cells showed a significant phenotypic improvement. To our knowledge, this is the first report of genetic correction of iPSC generated from somatic cells derived from an OTCD patient and, consequently, from cells harboring a pathogenic variant in the *OTC* gene. Specifically, the delivery of transgenes required for cell reprogramming was mediated through a Sendai virus vector, an advantageous reprogramming method because of its scarless nature generating stem cell clones free of genomic integration. Pluripotency was assessed through traditional methods, as well as PluriTest, a bioinformatic alternative which compares the transcriptomic profile of newly generated iPSC to those of well-documented stem cell lines [21,22]. Thereafter, characterized iPSC were subjected to genome engineering utilizing plasmids for gRNA delivery, and single stranded DNA donor templates. We opted for a clean, footprint-free method to avoid insertional mutagenesis, a hallmark concern of viral delivery. In addition, no selectable markers, such as antibiotic resistance genes or fluorescent markers, were introduced, maintaining the edited cells as close as possible to their natural properties. The endogenous gene was genetically corrected, instead of inserting the new functional gene, such as might be the case with traditional viral gene therapy; therefore, the expression of *OTC* continued to be controlled by endogenous promoters and enhancers, which otherwise might not be expressed in a cell-specific manner, and the possibility of viral gene silencing was eliminated. Although we show correction of one specific mutation in the *OTC* gene, more than 490 mutations are currently listed in the Human Gene Mutation Database [27], and the strategy proposed here could be easily tailored to other mutations by using the same primers to identify the disease-causing variants, and the subsequent design of suitable targeting guide RNA and donor templates. Finally, our lab has worked on hepatic differentiation, either from iPSC [28] or other cell types [29]. From our experience, as well as that documented by others [30,31,32], three-dimensional multicellular structures provide improved hepatic differentiation. Thus, we generated organoid iPSC-HLC following published protocols [33], as recently modified [28].

A combination of cell reprograming, genome editing and hepatic differentiation platforms could be exploited for therapeutic or research purposes, such as disease modeling, drug screening or as proof of concept for actual hepatic editing procedures in vivo. Standard care for patients affected by UCD includes limitation of protein intake and ammonia scavengers, which are only partially effective, and more prophylactic measures than curative [34]. In most severe cases, patients need to undergo liver or hepatocyte transplantation, which are based on the availability and compatibility of available donor organ or cells. Therefore, a promising therapeutic avenue for these types of diseases might evolve from the combination of stem cell and genome editing technologies, as described here.

A significant bottleneck for investigation, as well as identification and testing of novel therapeutics for treatment of liver diseases, is the inability to model the hepatic function and phenotype in vitro. Generation of iPSC-HLC could provide a platform for disease modelling and drug screening with a theoretically almost inexhaustible source of cells. Disease-specific iPSC can be obtained either from reprogrammed cells from patients with rare diseases, consequently retaining the genetic background, or be genetically modified with programmable nucleases. For instance, iPSC-HLC have been used for toxicity studies [35], or served as liver disease models [36].

However, the implementation of the aforementioned scenarios requires several obstacles to be overcome, with the most significant challenge being the poor differentiation of iPSC into hepatocytes. A number of factors—not fully understood—can affect the differentiation potential, including genetic background, age, the reprogrammed cell type, reprogramming method, passage number and epigenetic memory [37]. Despite many efforts in improving hepatic differentiation, iPSC-HLC resemble characteristics similar to fetal or newborn hepatocytes, rather than of adult primary counterparts. We recently published a methodological paper proposing a qPCR-based array to assess hepatic maturation, where our iPSC-HLC were compared to commercially available HLC, and although there were differences in maturation, all were characterized with residual fetal characteristics rather than full adult liver features [28]. Such immature levels of hepatic differentiation in several stem cell sources have been confirmed by others [38,39]. However, it is recognized that full mature hepatic differentiation may not be required, and that current differentiation protocols might be sufficient to recapitulate some disease phenotypes, as reported here. A broad range of pathologic conditions have been modelled using iPSC-derived hepatocytes [11]. The gene expression profile of unedited and edited iPSC-HLC in the present study indicates incomplete maturation, with fetal markers, such as alpha fetoprotein (AFP) and cytochrome P450 (CYP) 3A7, at high levels. Additionally, cells fail to reach mature levels of essential genes, such as human CYP450 genes, critical for the metabolism of endogenous and exogeneous compounds. Perhaps hepatic maturation could be improved with further research on 3D models, co-culture of iPSC-HLC with non-parenchymal cells, transplantation of generated cells and maturation in vivo, or investigation of factors that affect differentiation propensity of clones into specific lineages.

A number of studies have used CRISPR technology on iPSC attempting to repair defective genes related to hepatic pathologies. The majority show excellent molecular correction, but fail to prove fully functional phenotypic correction resembling mature primary hepatocytes. Specifically, some researchers have used fetal, instead of adult, hepatocytes for comparison, while others fail to show the rescue of phenotype [40,41]. In this study, the ratio of labelled to total urea produced by unedited and edited iPSC-HLC was quantified by mass spectrometry. A statistically significant difference in urea production between unedited and edited iPSC-HLC was measured. Thus, the correction of the genetic defect resulted in a verifiable phenotypic correction. While urea production by the HLC was still lower than that observed with mature hepatocytes, it was sufficient to verify genetic correction of the mutation. The low level of urea production in HLC compared to mature hepatocytes is most likely due to incomplete hepatic maturation, rather than improper genetic correction.

Unspecific and unintended genetic modification that can be caused by genome engineering applications is a major concern. Different methods are used to investigate off-target mutagenesis. Some techniques rely solely on in silico predictions by bioinformatic tools, such as Cas-OFFinder or CRISPR design. The detection is based on PCR amplification and sequencing or digestion with T7E1 or Surveyor assays of predicted loci. These techniques are considered biased due to the fact that they are based on a reference genome and rely on prediction software. Additionally, it has been reported that they do not accurately reflect the activity in edited cells [42]. On the other hand, other unbiased and more sophisticated methods, such as GUIDE-seq [43], CIRCLE-seq [44] and DISCOVER-seq [45], are based on deep sequencing and the patient’s own DNA instead of reference genomes, generating a personalized specificity profile. In our study, we opted for whole genome sequencing of unedited and edited cells to evaluate unintended mutagenesis, proving no off-targets in the genome of corrected cells. We consider this method to be suitable, since we are dealing with clones derived from single cells. In other circumstances where low detection limit would be a prerequisite, such as in the case of a mixed cell population of edited and unedited cells, the aforementioned detection methods based on targeted sequencing would be more appropriate.

In summary, this report describes the reprogramming, genetic correction and hepatic differentiation of somatic cells derived from an OTCD patient. The approach can undoubtedly be applied to other genetic liver diseases to investigate and rescue liver dysfunctions. Additionally, such technology may lay the foundation for the development and clinical evaluation of potential treatments for urea cycle disorders. Although it seems like an unlikely scenario, it is worth mentioning that parts of it are slowly being accomplished with iPSC, CRISPR and other programmable nucleases being currently tested in many clinical trials [46,47]. Advances in hepatic differentiation protocols, as well as further specificity and efficiency of programmable nucleases might make iPSC-HLC a suitable cell source for regenerative medicine and a valuable platform for drug testing and disease modeling in the future.

## 4. Materials and Methods

### 4.1. Cell origin and Culture

Liver fibroblasts and hepatocytes from a 9 month old OTC-deficient (OTCD) male donor, who underwent liver transplant, were collected following Ethical and Institutional Guidelines at the University of Pittsburgh under the IRB protocol 0411142. Hepatocyte isolation was performed as previously described [48]. All cells were maintained at 37 °C and 5% CO_2_ in a humidified incubator. Fibroblasts were cultured in a medium consisting of DMEM-GlutaMax (Thermo Fisher, Waltham, MA, USA) supplemented with 10% heat-inactivated fetal bovine serum (FBS) (Thermo Fisher, Waltham, MA, USA), 1 mM non-essential amino acids (NEAA; Thermo Fisher, Waltham, MA, USA) and 100 U/mL penicillin/streptomycin (Thermo Fisher, Waltham, MA, USA).

Induced pluripotent stem cells (iPSC) were cultured in Essential 8 medium (Thermo Fisher, Waltham, MA, USA) on vitronectin (Thermo Fisher, Waltham, MA, USA) coated dishes or plates. IPSC were mechanically passaged every 4–5 days when cultured as colonies. IPSC dissociated and cultured as single cells were passaged with Accutase (Sigma, St. Louis, MO, USA). Rock Inhibitor Y-27632 (10 μM, STEMCELL Technologies, Cambridge, UK) was added when cells seeded to prevent apoptosis.

Normal OTC-proficient (OTCP) hepatocytes, used for *OTC* transcript investigation, were procured at Karolinska Institute under the ethical approval Dnr. 2010/678-31/3. Hepatocytes were isolated from female donors, 68 and 78 years old, who underwent liver transplantation due to colorectal cancer metastasis. Cells were isolated from healthy regions of the liver.

### 4.2. DNA and RNA Isolation

Genomic DNA was extracted from cells with a DNeasy Blood and Tissue Kit (QIAGEN, Hilden, Germany) according to the manufacturer’s instructions. RNA extraction was performed either with a PureLink RNA Mini Kit (Thermo Fisher, Waltham, MA, USA) or by disruption of cells with TRIzol reagent (Thermo Fisher, Waltham, MA, USA) followed by separation of RNA from the water phase by isopropanol precipitation. DNA and RNA concentrations were quantified with a NanoDrop Spectrophotometer (Thermo Fisher, Waltham, MA, USA).

### 4.3. Long-Range PCR Amplification and Sequencing

Long range PCR was performed with PrimeSTAR GXL DNA polymerase (Takara, Kusatsu, Japan) according to the manufacturer’s instructions. Primers used for the amplification of the 8 regions are listed in Table S5. PCR amplicons were run on 0.5% agarose gel and extracted with QIAquick Gel Extraction Kit (QIAGEN, Hilden, Germany). Amplicons were pooled and target sequenced by Macrogen, Seoul, South Korea (Run type 100bp PE, platform HiSeq2000, Shotgun library type, TruSeq DNA PCR free 350bp). Reads were aligned to reference *OTC* gene on NCBI (Gene ID: 5009) with Geneious 8.1.9 (Geneious, Auckland, New Zealand).

### 4.4. Reprogramming

OTCD fibroblasts were derived from liver cells and reprogrammed with a Sendai-vector based reprogramming kit (CytoTune 2.0 Sendai Reprogramming Kit, Thermo Fisher, Waltham, MA, USA) delivering the Yamanaka factors [16], as previously described [49].

### 4.5. Gene Expression Analysis

Quantitative real-time PCR (qPCR) assay was conducted following a previously published protocol [28]. Specifically, a StepOnePlus real-time PCR system (Applied Biosystems, Foster City, CA, USA) was used for the amplification. RNA was isolated with a PureLink RNA Mini Kit according to the manufacturer’s instructions and concentration quantified with NanoDrop spectrophotometer. Complementary DNA was synthesized with High-Capacity cDNA Reverse Transcription Kit (Thermo Fisher, Waltham, MA, USA). TaqMan assays were used to quantify the gene expression, and they are listed in Table S6. Reactions were run in duplicates or triplicates, with Cyclophilin A (*PPIA*) as endogenous control in all experiments. Relative gene expression values were calculated according to the comparative method 2^(−ΔCt),^ where ΔCt = Ct gene of interest—Ct internal control Cyclophilin A. The cycle threshold was set to 0.082963 in all experiments, which was well within the linear range of the amplification curves. Ct values of interest 35 or higher were considered as unreliable, while those assigned as “undetectable” were set as Ct equal to 40.

### 4.6. Flow Cytometry

IPSCs cells were incubated with Accutase enzyme (Sigma, St. Louis, MO, USA) for 5 min at 37 °C in order to detach and make them single cells. Surface antigen staining: Cells were washed with PBS and stained for 20 min at room temperature with a Live Dead Violet Kit (Thermo Fisher, Waltham, MA, USA) and conjugated antibodies. PE-conjugated rat was conjugated with anti-SSEA3 (560237) and BV605-conjugated mouse anti-TRA1-60 (563187) (all from Becton Dickinson, Franklin Lakes, NJ, USA). Nuclear staining: Fixation and permeabilization was performed with Fix/Perm Buffer Set (ThermoFisher, Waltham, MA, USA) following the manufacturer’s instructions. Antibodies used were PerCP-Cy5.5-conjugated anti-OCT3/4 (560794), Alexa 647-conjugated anti-SOX2 (560302) and PE-conjugated anti-NANOG (560483) (all from Becton Dickinson, Franklin Lakes, NJ, USA). Cells were analyzed with an LSR Fortessa Analyzer (Becton Dickinson, Franklin Lakes, NJ, USA) and data with FCS Express 4 (De Novo Software, Pasadena, CA, USA). Isotype control was used in each case for gate setting.

### 4.7. Alkaline Phosphatase Staining and Immunocytochemistry

Alkaline phosphatase staining was performed using an Alkaline Phosphatase Live Stain Kit (Thermo Fisher, Waltham, MA, USA) and following the manufacturer’s instructions. IPSCs colonies were visualized with Olympus iX73 (Olympus, Tokyo, Japan).

### 4.8. PluriTest

RNA samples were isolated from fibroblasts or stem cells as described above. PluriTest was entirely performed at Mutation Analysis Facility (Karolinska Institute, Stockholm, Sweden). RNA integrity was analyzed with Agilent RNA 6000 Nano chips (Agilent Technologies, Santa Clara, CA, USA). RNA was amplified using an Illumina Total Prep RNA Amplification Kit (Ambion, Austin, TX, USA) according to the published protocol [21,22]. The assay was performed by profiling whole genome expression using Human HT-12 v4 Expression Bead Chip Array (Illumina, San Diego, CA, USA). Thereafter, the array was compared with data in the Stem Cell Matrix 2 Database (SCM2). Analysis of sequencing data and representation on SCM2 database was performed with bioinformatic tools provided at www.pluritest.org (Stockholm, Sweden).

### 4.9. gRNA Design

A previously published protocol for the application of CRISPR/Cas9 was followed and modified for the mutation and the cells used in this study [50]. Guide RNA was designed against the human *OTC* gene, targeting as close as possible to the disease-causing variant, using the online CRISPR Design tool (http://tools.genome–engineering.org, Cambridge, MA, USA). Two gRNAs were designed in order to be used with the wild-type Cas9 nuclease (pX458 vectors, Addgene, Watertown, MA, USA), and two pairs of gRNAs to be used with D10A nickase mutant version Cas9 (Cas9 nickase, Cas9n, vector pX461, Addgene, Watertown, MA, USA), as shown in Figure 3a. Vectors expressed wild-type SpCas9 or D10A nickase SpCas9, tracrRNA and Green Fluorescent Protein (GFP), used for sorting positive-transfected cells. DNA sequences of gRNAs to be cloned into the above vectors were ordered from Sigma, St. Louis, MO, USA. If the first base of the gRNA sequence was not a guanidine, then a guanidine on position 21 was added for optimal expression of human U6 promoter. Additionally, 3′- and 5′-overhangs were added as shown in Table S1 to facilitate the cloning.

### 4.10. Vector Construction

DNA sequences were ordered from Sigma, St. Louis, MO, USA. For the cloning of gRNAs into the respective vector, 4.5 μL (100 μM) of the top strand, 4.5 μL (100 μM) of the bottom strand and 1 μL of 10x T4 DNA ligase buffer (BioLab, Lawrenceville, GA, USA) were incubated at 95 °C for 5 min and left to cool down at room temperature for 45 min. Sequences of gRNAs are shown in Table S1.

For the insertion of gRNA into pX458/pX461 vectors, 2 μL of 10x Fast Digestion Buffer (Fermentas, Waltham, MA, USA), 15 μL water, 1000 ng pX458/pX461 and 1 μL of Bpil (BbsI 10 U/μL, ThermoFisher, Waltham, MA, USA) were incubated at 37 °C for 1 h. Next, 2.5 μL of 10 x T4 DNA ligase buffer (BioLab, Lawrenceville, GA, USA), 1 μL annealed oligos and 1.5 μL T4 DNA ligase (BioLab, Lawrenceville, GA, USA) were mixed with the digestion reaction and incubated at 37 °C. For the transformation, 2 μL of ligated samples was mixed with 25 μL One Shot^®^ Stbl3™ Chemically Competent Escherichia coli (Thermo Fisher, Waltham, MA, USA) and left on ice for 30 min, followed by heat shock at 42 °C for 45 s, and finally incubated on ice for 2 min.

For the culture of transformed bacteria, 250 μL pre-warmed SOC Outgrowth Medium (BioLab, Lawrenceville, GA, USA) was added to each sample and incubated at 37 °C for 1 h at 225 rpm. Around 100 μL of the mixture was placed on LB agar ampicillin plates and incubated overnight at 37 °C. Extraction of plasmid was performed with a QIAprep Spin Miniprep Kit (QIAGEN, Hilden, Germany) according to the manufacturer’s instructions, and concentration was quantified with NanoDrop spectrophotometer.

In order to test the successful insertion of gRNA into the respective vector, 1 μL (500 ng/μL) of each sample was digested with 0.5 μL Bpil (BbsI, 10 U/μL, Thermo Fisher, Waltham, MA, USA) and 0.5 μL BshTI (AgeI, 10 U/μL, Thermo Fisher, Waltham, MA, USA). The DNA was run on 1.5% agarose gel. The successfully digested products were Sanger-sequenced (Macrogen, Seoul, Korea) to ensure that the gRNA sequence was inserted.

### 4.11. Transfection

Cells were treated with 10 μM Rock Inhibitor Y-27632 for 3 h before transfection. IPSC were transfected using a 4D-Nucleofector System (Lonza, Basel, Switzerland) with an electroporation buffer P3 Primary Cell 4D-Nucleofector X Kit L (Lonza, Basel, Switzerland) and program CA137. Cells were detached with Accutase and washed with PBS. Around 8 × 10^5^ cells were mixed with either 7 μg pX358 vector expressing wild-type SpCas9, tracrRNA and gRNA or 3.5 μg of pX461 vector expressing D10A nickase SpCas9, tracrRNA and one gRNA and 3.5 μg of pX461 vector expressing D10A nickase SpCas9, tracrRNA and the other gRNA of the pair to be used with the nickase approach. Donor templates were used as single stranded DNA named DT7.1 for wild-type Cas9-gRNA1 and nickase Cas9-gRNA3a-gRNA3b, or DT7.23 for wild-type Cas9 gRNA2 and nickase Cas9-gRNA4a-gRNA4b. For each reaction, 2 μL of 100 μΜ donor template was used. Sequences of donor template are provided in Supplementary Table S4.

The format of transfection was NucleocuvetteTM Vessels (Lonza, Basel, Switzerland). Each reaction was seeded in one well of 24-well plate vitronectin in mTeSR medium supplemented with 10 μM Rock Inhibitor Y-27632. Cells were enriched based on GFP positivity 24 h post transfection, as described below.

### 4.12. Fluorescence-Activated Cell Sorting (FACS) of Transfected Cells

Enrichment of transfected cells was performed 48 h post transfection based on GFP positivity. Cells were sorted using fluorescence-activated cell sorting (FACS) (BD, Franklin Lakes, NJ, USA FACSaria IIu machine, 100 μm needle and 20 pSi pressure). SYTOX (1 x, 1 μL per sample, Thermo Fisher, Waltham, MA, USA) was used to remove dead cells from the flow cytometry data. Cells were collected on vitronectin 96-well plates in Essential 8 medium, supplemented with 10 μM Rock Inhibitor Y-27632.

### 4.13. Restriction Enzyme Digestion and Analysis of DNA Fragments

HpaII restriction enzyme (NEB, Ipswich, MA, USA) was used for the evaluation of editing efficiency, only if the target had been successfully corrected (CCGG). Specifically, target locus was amplified with Pfu Ultra II Fusion HS DNA polymerase using 1 μL enzyme, 1 μL dNTPs (10 mM each), 1 μL forward primer (10 μM, 5′-ATGCCTCGTTATTGCCAGGT-3′), 1 μL reverse primer (10 μM, 5′-GGACCTTCATTTCCTCCTCAACT-3′), 5 μL 10x reaction buffer and 150 ng DNA for each reaction of final volume 50 μL. PCR product was treated with HpaII restriction enzyme by incubating 200 ng PCR product with 2 μL 10x CutSmart buffer, 1 μL enzyme for every reaction of 20 μL final volume followed by incubation at 37 °C for 45 min. Analysis of DNA fragments was performed on 1.5% agarose gel. Gel electrophoresis images were visualized with LI-COR ODYSSEY 2800 (LI-COR, Lincoln, NB, USA) and band intensities estimated with LI-COR Image Studio 3.1.4 software (LI-COR, Lincoln, NB, USA. Cleavage efficiencies were estimated based on the formulas below: f_cut_ = (b + c)/(a + b + c), where c is the intensity of wild-type band, while a and b are the intensities of the digested bands. Cleavage (%) = 100 × (1 − √(1 − *f*cut)) [50]. Moreover, homology-directed repair (HDR) efficiency was further validated with Synthego Performance Analysis, Interference of CRISPR Edits (ICE) Analysis. 2019. v2.0. Synthego (https://ice.synthego.com, Redwood, CA, USA). Specifically, PCR amplicons of the region of interest (from unedited and edited cells) were Sanger-sequenced (Macrogen, Seoul, Korea) and analyzed with the ICE web tool.

### 4.14. Single Cell Clonal Expansion and Editing Verification

Cells were serially diluted to a concentration of 0.5 cells/100 μL mTeSR supplemented with 10 μM ROCK inhibitor and seeded 100 μL per well in a vitronectin coated 96-well plate. The medium was changed daily. Clones were expanded and analyzed as described in the “Restriction enzyme digestion and analysis of DNA fragment” method section. Fully digested clone was Sanger-sequenced (Macrogen, Seoul, Korea) in order to verify the successful genetic correction.

### 4.15. OTC Transcript Amplification

Total RNA isolated either from primary hepatocytes (OTCD or OTCP), or genetically edited or unedited iPSC-HLC was isolated with PureLink RNA extraction kit. Complementary DNA was synthesized with PrimeScript Reverse Transcriptase (Takara, Kusatsu, Japan) according to the manufacturer’s instructions. *OTC* mRNA was amplified using Pfu Ultra II Fusion HS DNA polymerase from exon 1 to exon 5 with forward primer 5′-GAAGATGCTGTTTAATCTGAGG and reverse primer 5′-CTGGAGCGTGAGGTAATCAGCC, and from exon 5 to exon 10 with forward primer 5′-GCAGATGCAGTATTGGCTCG and reverse primer 5′-CCCATACCACGTGTTAGGGATT, as previously described [24]. Produced products were visualized on 1.5% agarose gel and Sanger-sequenced (Macrogen, Seoul, Korea). Geneious 8.1.9 software was used for sequence view, alignment and illustration, as well as for PCR primer design.

### 4.16. Definitive Endoderm Optimization

Two different definitive endoderm protocols were tested initially in order to select the iPSC clone with the highest differentiation propensity into hepatocytes. Protocol DE-A contains on days 1 and 2 RPMI medium (Thermo Fisher, Waltham, MA, USA supplemented with 1% B27 (Thermo Fisher, Waltham, MA, USA), 1% NEAA, 100 U/mL penicillin/streptomycin, 100 ng/mL ActivinA (PeproTech, Cranbury, NJ, USA). On days 3 and 4, the above media was supplemented with 10ng/mL bFGF (PeproTech, Cranbury, NJ, USA) and 20 ng/mL BMP4 (PeproTech, Cranbury, NJ, USA). Protocol DE-B contains RPMI, 1% B27, 1% NEAA, 100 U/mL penicillin/streptomycin, 50 ng/mL ActivinA, 2.5 μΜ CHIR (only on day 1) (STEMCELL Technologies, Cambridge, UK), as well as Knock-Out serum replacement (KOSR; ThermoFisher, Waltham, MA, USA) and Insulin-Transferrin-Selenium 100x (ITS; Thermo Fisher, Waltham, MA, USA)—each of those 0.1% on day 1, 1% on day 2, 2.5% on days 3 and 4. Both protocols were performed in 6-well plates coated with Matrigel (BD, Franklin Lakes, NJ, USA) (0.67 mg/well). Hepatic differentiation: The protocol for hepatic differentiation is described in a previous study from our laboratory [28]. Culture and seeding: IPSC were routinely cultured in Essential 8 medium on vitronectin-coated dishes. Cells were detached as single cells using Accutase and seeded 10^5^ per well of 6-well Matrigel coated plate (0.67 mg/well). Definitive endoderm induction—days 1–4: Protocol DE-B, described above, was used for the hepatic differentiation. Hepatoblast formation—days 5–6: RPMI GlutaMax medium, 1% NEAA, 100 U/mL penicillin/streptomycin, 1% B27, 10 ng/mL FGF and 20 ng/mL BMP4. Organoid formation—days 7–13: Cells were harvested on day 8 with Accutase enzyme, and 5 × 105 cells were resuspended in 300 μL Matrigel and plated in 6-well plates. Organoid medium was prepared as previously described [25] without the addition of forskolin and replacement of Wnt3a and R-spondin 1 conditioned media with 33 ng/mL Wnt3a protein (PeproTech, Cranbury, NJ, USA) and 0.66 μg/mL protein R-spondin 1 (PeproTech, Cranbury, NJ, USA). Hepatic differentiation—days 14–26: The medium of the last phase of hepatic differentiation consisted of 45% Ham’s F12 Nutrient Mix medium (Thermo Fisher, Waltham, MA, USA), 45% DMEM GlutaMax Low Glucose medium (Thermo Fisher, Waltham, MA, USA), 10% KOSR, 1% NEAA, 100 U/mL penicillin/streptomycin, 20 ng/mL HGF (PeproTech, Cranbury, NJ, USA), 5 mM rifampicin (Sigma, St. Louis, MO, USA), 10 mM hydrocortisone 21-hemisuccinate sodium salt (Sigma, St. Louis, MO, USA), 0.1 mM ascorbic acid (Sigma, St. Louis, MO, USA), 10 mM lithocholic acid (Sigma, St. Louis, MO, USA), 10 mM vitamin K2 (Sigma, St. Louis, MO, USA), 10 ng/mL human growth hormone (Sigma, St. Louis, MO, USA), 0.1 mM dexamethasone (Lonza, Basel, Switzerland) and 10 ng/mL oncostatin M (PeproTech, Cranbury, NJ, USA), and differentiation medium was supplemented with 1 mM 8-Bromoadenosine 3′5′-cyclic monophosphate sodium salt (Sigma, St. Louis, MO, USA) for the last 4 days.

### 4.17. Enzyme-Linked Immunosorbent Assay (ELISA)

Secreted albumin in the medium was quantified on days 20 and 26 of hepatic differentiation in biological triplicates and technical duplicates using a Quantitative Human Albumin ELISA Quantitation Kit (Bethyl Laboratory, Montgomery, TX, USA). Albumin was normalized to DNA amount extracted from the cells from the respective well, as well as the incubation time (22 h) at day 20 and 26 of hepatic differentiation.

### 4.18. In Vitro ^15^N-Labelled Urea Production

Genetically corrected or uncorrected iPSC-HLC were incubated in differentiation medium with 1 mM ornithine (Sigma, St. Louis, MO, USA) and 1 mM ^15^NH_4_Cl (Sigma, St. Louis, MO, USA) for 22 h on days 20 and 25. Media were collected and unlabeled (m/z 231), and labelled urea (m/z 332 and 233) was quantified with mass spectrometry as previously described. Samples were deproteinized with cold acetone (VWR, Radnor, PA, USA), centrifuged and cleaned on 1 mL AG-50W-X8 resin (analytical grade 100–200 mesh, hydrogen form, BioRad, Hercules, CA, USA). Afterwards, samples were dried in a rotary evaporator (Speedvac, ThermoFisher, Waltham, MA, USA) and derivatized with ethyl acetate (VWR, Radnor, PA, USA) and MTBSTFA (Biotech AB) (1/1) at 60 °C for 60 min. Analysis of mass to charge (m/z) was performed on an Agilent (Santa Clara, CA, USA) GC-MS System (Gas chromatograph 6890, Mass Spectrometer 5975) with a non-polar GC column (Agilent HP-5MS). M/z 231, 232, 233 were detected in electron ionization mode. The amount of labelled urea was expressed as molar percent excess (MPE) calculated from the ratio of labelled/unlabeled urea (Rs) [51].

### 4.19. Off-Target Evaluation

Off-target mutagenesis analysis was conducted by Macrogen, Seoul, South Korea. Whole genome sequencing was performed on genomic DNA from both genetically unedited and edited iPSC. Illumina-Shotgun, TruSeq DNA PCR-free (350 bp insert) (Illumina, San Diego, CA, USA) was used as the library for the next generation sequencing. Sequencing coverage was 30x and read length Illumina 150 bp PE. To identify potential off-targets, gVCF files were merged from sequencing results of unedited and edited cells, and differences were extracted. Run BLAST for target sequence: GAATGAAAGTCTCACGGACA with command: blastn -task blastn -word_size 7 -penalty -3 -query target_seq.fa\-subject gvcfmerge.refSeq.fasta -outfmt 6 -out off-target.txt. Potential off-target regions were viewed by IGV.

### 4.20. Illustrations

Illustrations in Figure 1a,b, Figure 2a,d and Figure S2 were generated with Geneious 8.1.9 software (Geneious, Auckland, New Zealand). Illustrations in Figure 1d and Graphical abstract were partly generated with images from ©Adobe Stock (Adobe, Mountain View, CA, USA) and edited with Illustrator 24.3 software (Adobe, Mountain View, CA, USA).

### 4.21. Statistical Analyses

All statistical analyses were performed with Graph Pad Prism (GraphPad, San Diego, CA, USA). Sample size (n) is indicated in the relevant figure legend. A two-tailed, unpaired Mann–Whitney U test was used to compare means between two experimental groups. The level of significance was set at *p*-value < 0.05 for all the experiments (summarized as ns: not significant; * *p* < 0.05; ** *p* < 0.01).

## Data Availability

The data presented in this study are available in this paper and supplementary files.

## Supplementary Materials

The following are available online at https://www.mdpi.com/1422-0067/22/3/1217/s1, Figure S1: Verification of homology-directed repair (HDR)-mediated editing efficiency. Figure S2: Sequencing of *OTC* transcript. Figure S3: Comparison of pluripotency between unedited and edited iPSC clones. Figure S4: Selection of iPSC clone and protocol for definitive endoderm induction. Figure S5: Optimization of seeding density and coating material for definitive endoderm induction. Figure S6: Morphology of organoid iPSC hepatocyte-like cells (iPSC-HLC). Figure S7: Gene expression profiling of organoid iPSC hepatocyte-like cells (iPSC-HLC). Table S1: gRNA sequences. Table S2: DNA donor template sequences. Table S3: Variant list in off-target regions. Table S4: Variant list in on-target region. Table S5: Sequences of primers used for long-range PCR amplification. Table S6: TaqMan assays used for gene expression analyses.
