# Supplementary material for: Gene Editing Correction of a Urea Cycle Defect in Organoid Stem Cell Derived Hepatocyte-like Cells
Source: Int J Mol Sci. 2021 Jan 26;22(3):1217. doi: 10.3390/ijms22031217 (PMC7865883; doi:10.3390/ijms22031217)
Supplement: Supplementary file 1 [file ijms-22-01217-s001.pdf]

Supplementary Material

Article

Gene Editing Correction of a Urea Cycle Defect in Organoid Stem Cell Derived Hepatocyte-like Cells

Mihaela Zabulica [1], Tomas Jakobsson [1], Francesco Ravaioli [2], Massoud Vosough [3], Roberto Gramignoli [1], Ewa Ellis [4], Olav Rooyackers [4] and Stephen C. Strom [1,*]

[1] Department of Laboratory Medicine, Karolinska Institute, 141 52 Stockholm, Sweden; mihaela.zabulica@ki.se (M.Z.); tomas.jakobsson@ki.se (T.J.); roberto.gramignoli@ki.se (R.G.)

[2] Department of Experimental, Diagnostic and Specialty Medicine, University of Bologna, 40 138 Bologna, Italy; francesco.ravaioli2@unibo.it

[3] Department of Regenerative Medicine, Cell Science Research Centre, Royan Institute for Stem Cell Biology, Tehran 16635-148, Iran; masvos@royaninstitute.org

[4] Department of Clinical Sciences Intervention and Technology, Karolinska Institute, 141 86 Stockholm, Sweden; ewa.ellis@ki.se (E.E.); olav.rooyackers@ki.se (O.R.)

* Correspondence: stephen.strom7.4@gmail.com

Citation: Zabulica, M.; Jakobsson, T.; Ravaioli, F.; Vosough, M.; Gramignoli, R.; Ellis, E.; Rooyackers, O.; Strom, S.C. Gene editing correction of a urea cycle defect in organoid stem cell derived hepatocyte-like cells. *Int. J. Mol. Sci.* **2021**, *22*, 1217. https://doi.org/10.3390/ijms22031217

Received: 30 December 2020
Accepted: 20 January 2021
Published: 26 January 2021

Publisher's Note: MDPI stays neutral with regard to jurisdictional claims in published maps and institutional affiliations.

Copyright: © 2021 by the authors. Licensee MDPI, Basel, Switzerland. This article is an open access article distributed under the terms and conditions of the Creative Commons Attribution (CC BY) license (http://creativecommons.org/licenses/by/4.0/).

Supplementary Figures

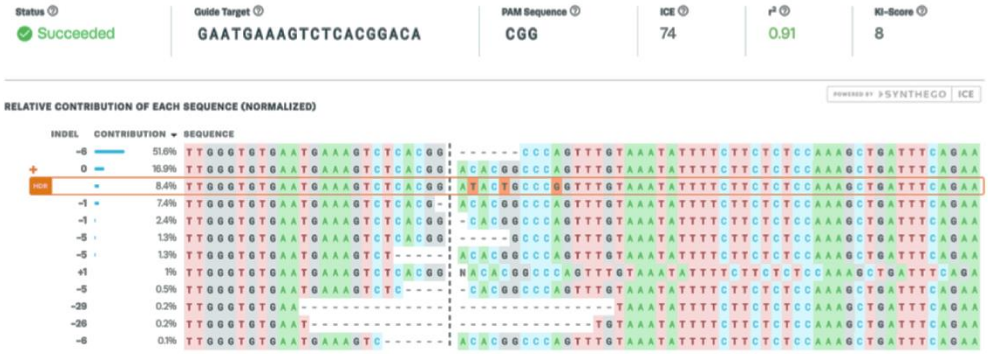

Figure S1: Verification of homology-directed repair (HDR)-mediated editing efficiency. HDR-mediated editing efficiency was validated with the web-tool Inference of CRISPR Edits (ICE, Synthego), along with the percentages of insertions/deletions of various lengths. The platform estimated the HDR efficiency to be 8.4%.

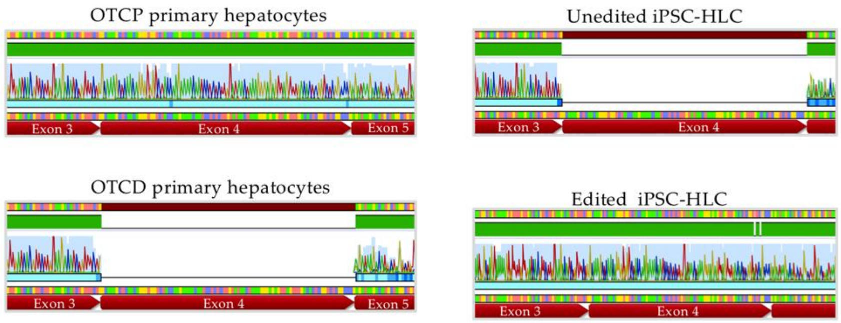

Figure S2: Sequencing of *OTC* transcript. *OTC* transcript in OTC-proficient (OTCP) and OTC-deficient (OTCD) primary hepatocytes, as well as in unedited and edited iPSC hepatocyte-like cells (iPSC-HLC) were sequenced and aligned to reference *OTC* transcript (NCBI). Transcripts in OTCD primary hepatocytes and unedited iPSC-HLC are lacking exon 4, while the exon is present in edited iPSC-HLC, same as in OTCP primary cells.

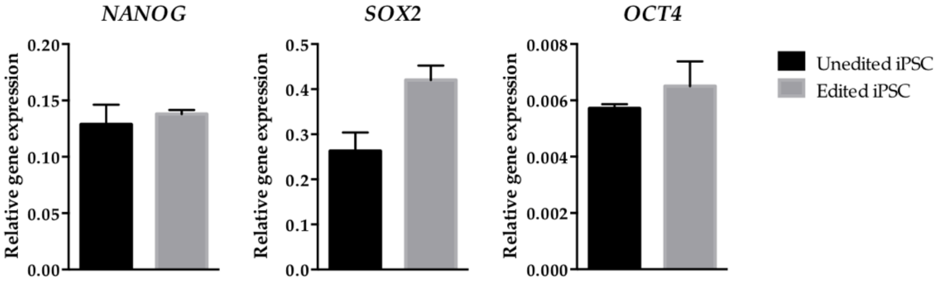

Figure S3: Comparison of pluripotency marker levels between unedited and edited iPSC clones. Gene expression of pluripotency markers was assessed in unedited and genetically edited iPSC clones and normalized to endogenous gene (*PPIA*). Technical replicates n=2.

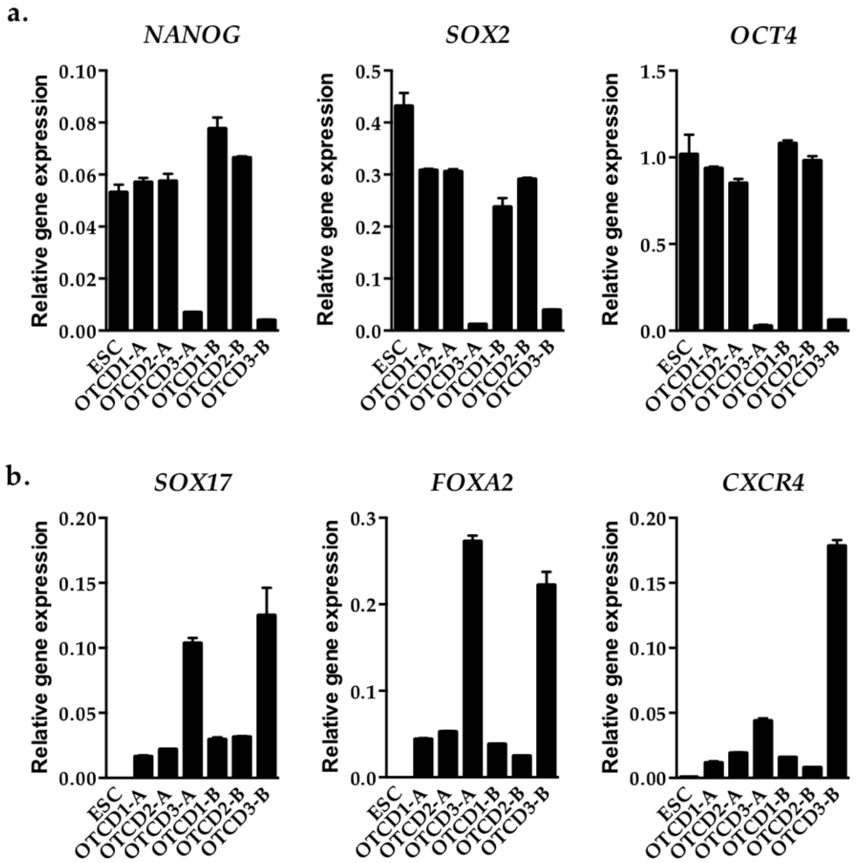

Figure S4: Selection of iPSC clone and protocol for definitive endoderm induction. Three iPSC clones (OTCD1, OTCD2 and OTCD3) were submitted to endoderm differentiation with DE protocol A or DE protocol B. **(a)** Expression of pluripotency (*NANOG*, *OCT4* and *SOX2*) and **(b)** definitive endoderm genes (*SOX17*, *FOXA2* and *CXCR4*) was analyzed and compared to the respective levels in undifferentiated embryonic stem cells (ESC). Gene expression was normalized to endogenous gene (*PPIA*). Technical replicates n=3.

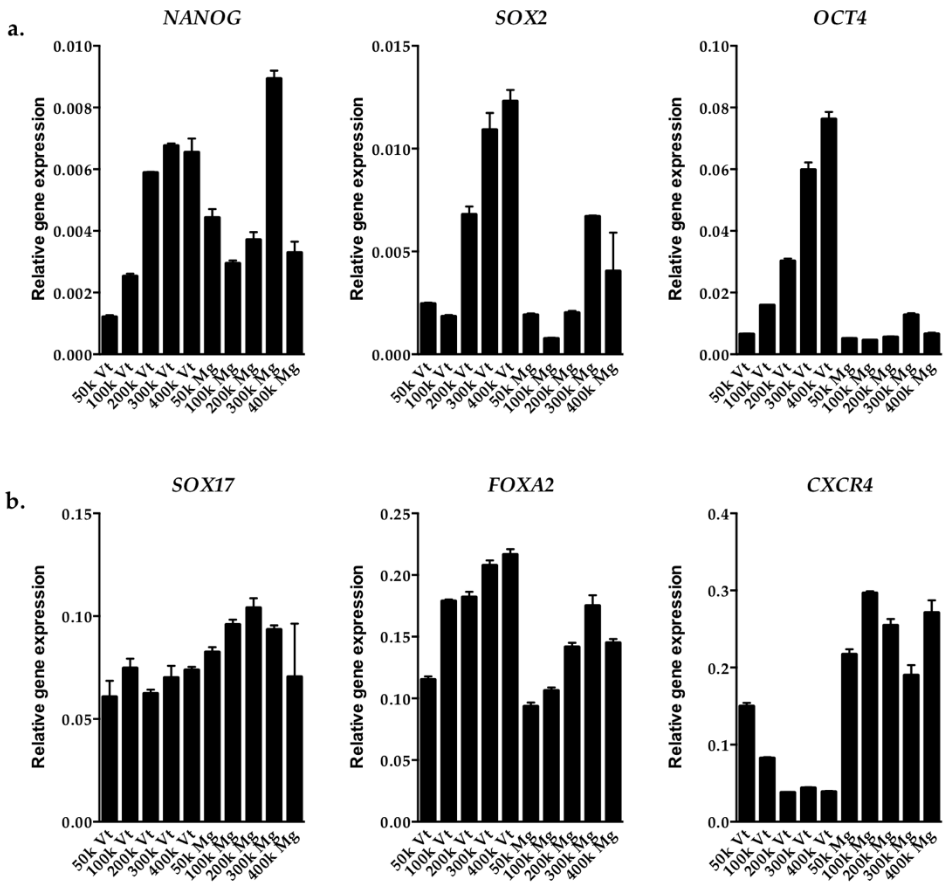

Figure S5: Optimization of cell seeding density and coating material for definitive endoderm induction. Optimization experiments were performed to identify the optimal cell seeding density (50k, 100k, 200k, and 400k) and coating material (Vt: Vitronectin and Mg: Matrigel) for definitive endoderm differentiation. Cells were submitted to definitive endoderm induction with DE protocol B. The efficiency of induction was determined through gene expression levels of essential pluripotency **(a)** and definitive endoderm genes **(b)**. Expression was normalized to endogenous gene control (*PPIA*). Technical replicates n=3.

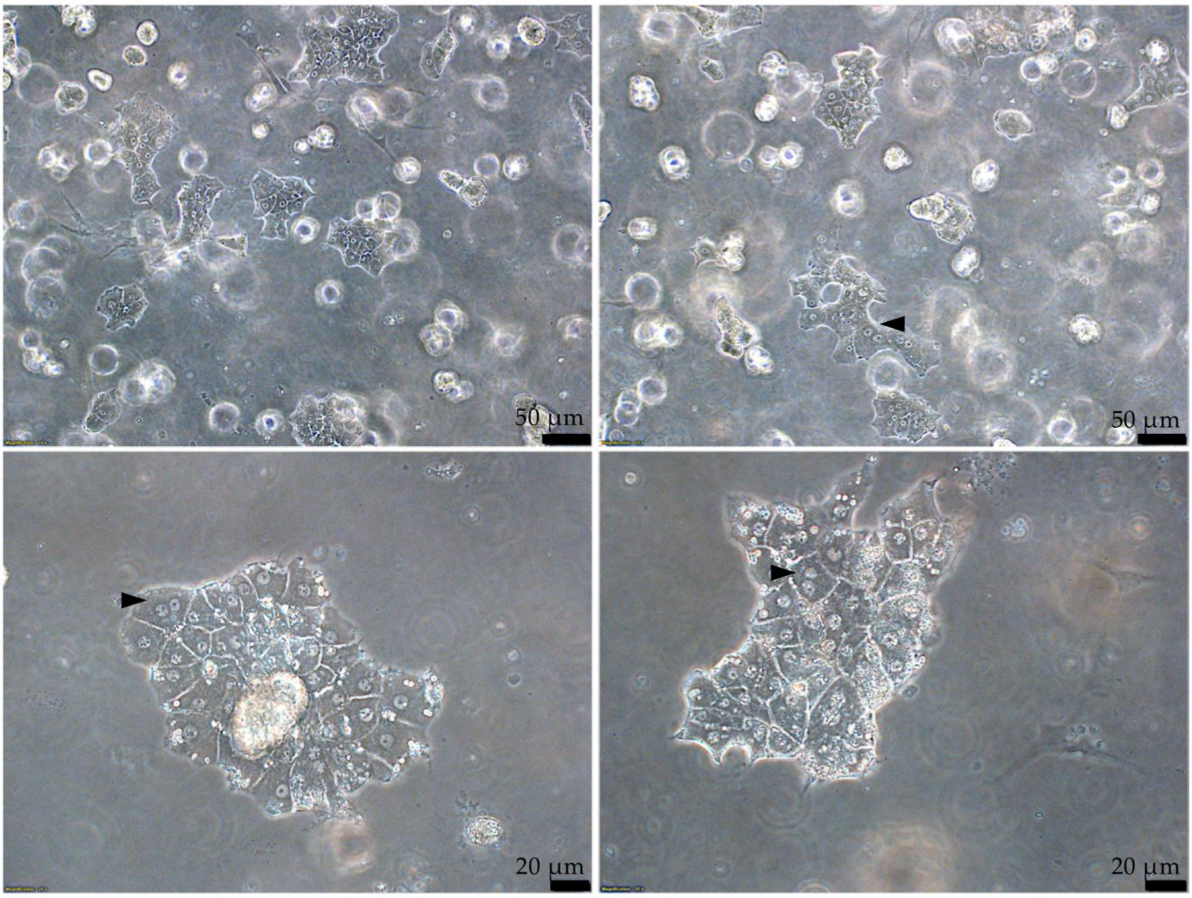

Figure S6: Morphology of organoid iPSC hepatocyte-like cells (iPS-HLC). Representative pictures of organoid iPSC-HLC which eventually seeded at the bottom of the culture plate, facilitating the visualization of the morphology, are presented. Spare binucleated iPSC-HLC were observed (indicated with arrows). Scale bar indicated in each image.

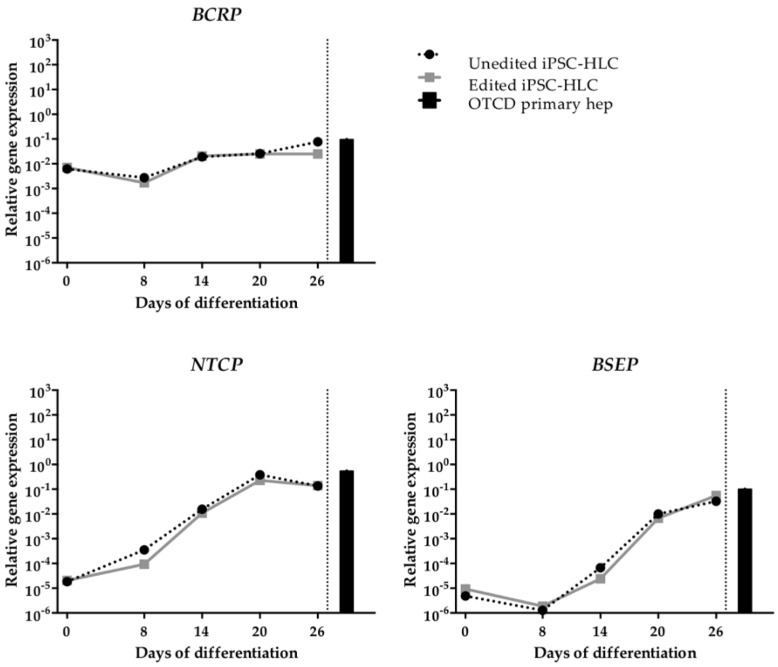

Figure S7: Gene expression profiling of organoid iPSC hepatocyte-like cells (iPSC-HLC). Expression of genes encoding transporter proteins was measured at different time points of differentiation protocol. Dashed and continuous lines show unedited and edited iPSC-HLC, respectively. Black bar indicates the level of expression of the respective gene in primary OTCD hepatocytes from the same patient. Expression levels were normalized to endogenous gene (*PPIA*).

Supplementary Tables

Table S1: gRNA sequences.

gRNA name	Vector	Sequence gRNA on + DNA strand 5′-3′ *	Sequence gRNA on + DNA strand 5′-3′ *
1	pX458 Wild type Cas9	caccGAAAGTCTCACGGACAC-GGCC	aaacGGCCGTGTCCGTGA-GACTTTC
2	pX458 Wild type Cas9	caccGAATGAAAGTCTCACGGACA	aaacTGTCCGTGAGACTTTCATTC
3a	pX461 D10A nickase Cas9	caccGAAAGTCTCACGGACAC-GGCC	aaacGGCCGTGTCCGTGA-GACTTTC
3b	pX461 D10A nickase Cas9	caccGCACAAGATATTCATTTGGGT	aaacACCCAAATGAATATCTT-GTGC
4a	pX461 D10A nickase Cas9	caccGAATGAAAGTCTCACGGACA	aaacTGTCCGTGAGACTTTCATTC
4b	pX461 D10A nickase Cas9	caccGTGTTTTCTTACCACACAAGA	aaacTCTTGTGTGGTAA-GAAAACAC

** Note: Additional nucleotides were added for the cloning (red, lowercase) and a guanidine (G) in some cases where the position 20 was not a G, in order to enhance the expression by the human U6 promoter.*

Table S2: DNA donor template sequences.

Donor template name	Used for gRNAs	Sequence 5′-3′
DT 7.1	pX458 – Wild type Cas9 with gRNA1<br><br>pX461 – D10A nickase Cas9 with gRNA3a and gRNA3b	AAAGAGAATTATGTTTTATTTTTGAAATTTATCCATCAGATTCTGAAATCAGCTTTGGAGAGAAGAAAATATTTACAAACCGGGCAGTATCCGTGAGACTTTCATTCACACCCAAATGAATATCTTGAGTCGTAAGAAAACAAGGATGTCCTCCCAGAAGTGC
DT. 7.23	pX458 – Wild type Cas9 with gRNA2<br><br>pX461 – D10A nickase Cas9 with gRNA4a and gRNA4b	AAAGAGAATTATGTTTTATTTTTGAAATTTATCCATCAGATTCTGAAATCAGCTTTGGAGAGAAGAAAATATTTACAAACCGGGCAGTATCCGTGAGACTTTCATTCACACCCAAATGAATATCTTGTGTGGTAAGGAAGCACGGATGTCCTCCCAGAAGTGC

Table S3: Variant list in off-target regions.

Chromosome				Unedited Cells			Edited Cells							
Chr	Position	Ref	Alt	Ref depth	Alt depth	Genotype	Ref depth	Alt depth	Genotype	Effect	Amino acid change	Codon change	Gene	dsSNP142
chr5	114940200	C	A	20	23	0/1	24	22	0/1	Intron	-	-	TMED7-TICAM2	rs10079000
chr11	112978377	G	A	0	31	1/1	0	39	1/1	Intron	-	-	NCAM1	rs12788208
chr13	87536313	G	A	18	13	0/1	16	18	0/1	Intergenic	-	-	-	rs9301816

Table S4: Variant list in on-target region.

Chromosome				Unedited Cells			Edited Cells							
Chr	Position	Ref	Alt	Ref depth	Alt depth	Genotype	Ref depth	Alt depth	Genotype	Effect	Amino acid change	Codon change	Gene	dsSNP142
chrX	38240674	C	T	15	0	0/0	0	18	1/1	Synonymous coding	gaC/gaT	D126	OTC	-
chrX	38240677	G	T	16	0	0/0	0	18	1/1	Synonymous coding	agC/acT	T127	OTC	-
chrX	38240682	G	A	0	16	1/1	19	0	0/0	Non-synonymous coding	cGt/cAt	R129H	OTC	rs66656800

Table S5: Sequences of primers used for long range PCR amplification.

Region	Forward primer<br>5′- sequence - 3′	Reverse primer<br>5′- sequence - 3′
1	CCCACACAGCACAGAGGATT	GGTGGTGAGGGCCTGTAATC
2	ACCGCTTTTGTCCCCAAGAA	GAGTCTGAATGTGGGGTGGG
3	TGAGCCAAGATCGTGCCATT	GGCGAGAGAGTGAGACTTCG
4	AGCTGCTACCACCTTGTTCC	CTCAGTGCTCCCCTTCAGTC
5	AATGTTCTCCGCGTTCGTTC	ATCCGGCCTCAGTCTACCAA
6	CTTCTTGGGCAGCCAGATGA	CCAGAAGCAGGAAGAGGCAA
7	ACCTTGCTGCTCTCTCACAG	GGCAACATGATGAAACCCCC
8	TAGCTGCAAGGGAGTTTGGG	AGAATTGGGCTGAGAATGAGGT

Table S6: TaqMan assays used for gene expression analyses.

	Gene name	Gene full name	Assay ID
	PPIA	Cyclophilin A (peptidylprolyl isomerase A)	Hs99999904_m1
Urea cycle genes	*OTC*	Ornithine carbamoyltransferase	Hs00166892_m1
	CPS1	Carbamoyl-phosphate synthetase 1, mitochondrial	Hs00157048_m1
	ASS1	Argininosuccinate synthetase	Hs01597989_g1
	ASL	Argininosuccinate lyase	Hs00902699_m1
	ARG1	Arginase 1	Hs00163660_m1
Liver proteins and transcription factors	*ALB*	Albumin	Hs00609411_m1
	AFP	Alpha-fetoprotein	Hs00173490_m1
	FAH	Fumarylacetoacetate hydrolase	Hs00908445_m1
	A1AT	Alpha-1 antitrypsin (SERPINA1)	Hs01097800_m1
	HNF4a (total)	Hepatic nuclear factor 4 alpha	Hs00230853_m1
	HNF4a (fetal isoforms)	Hepatic nuclear factor 4 alpha	Hs01025522_m1
	HNF4a (adult isoforms)	Hepatic nuclear factor 4 alpha	Hs00604431_m1
	HNF3a	Hepatic nuclear factor 3 alpha	Hs04187555_m1
	HNF3b	Hepatic nuclear factor 3 beta	Hs00232764_m1
	FXR	Farnesoid X nuclear receptor (NR1H4)	Hs00231968_m1
Phase I and II genes	*CY1A2*	Cytochrome P450 family 1 subfamily A member 2	Hs01070374_m1
	CYP2B6	Cytochrome P450 family 2 subfamily B member 6	Hs03044634_m1
	CYP3A4	Cytochrome P450 family 3 subfamily A member 4	Hs00430021_m1
	CYP3A7	Cytochrome P450 family 3 subfamily A member 7	Hs00426361_m1
	UGT1A6	UDP glucuronosyltransferase 1 family, A6	Hs01592477_m1
Pluripotency genes	*NANOG*	Nanog homeobox	Hs04260366_g1
	OCT4	Octamer-binding transcription factor	Hs00742896_s1
	SOX2	SRY (sex determining region Y)-box 2	Hs01053049_s1
	CXCR4	C-X-C receptor type 4	Hs00607978_s1
	SOX17	SRY (sex determining region Y)-box 17	Hs00751752_s1
Transporters	*BSEP*	ATP-binding cassette, B11 (ABCB11)	Hs00184824_m1
	BCRP	ATP-binding cassette, G2 (ABCG2)	Hs00184979_m1
	NTCP	Sodium/bile acid cotransporter 1 (SLC10A1)	Hs00914889_m1
